# Identifying Small Molecule Compounds that Have Synergistic Effects With Cisplatin and Other Platinum Drugs Using High Throughput Matrix Screening

**DOI:** 10.1200/GO.22.63000

**Published:** 2022-05-05

**Authors:** Layla Al-Zubi, Dorian M. Cheff, Zina Itkin, Hui Guo, Min Shen, Matthew D. Hall

**Affiliations:** ^1^ National Center for Advancing Translational Science, National Institutes of Health, Bethesda, MD

## Abstract

High grade serous ovarian cancer (“HGSOC”) is the seventh most common cancer worldwide among women. The disease has no unique symptoms and often women are diagnosed in advance stages. The World Health Organization estimates that 225,500 women are diagnosed and 140,200 will die of this disease per year. In the United States, the 5-year survival rate is 43%, and 85% of all HGSOC patients experience recurrence despite aggressive treatment. More efficacious drug combination treatments are needed. Recently it was found that platinum drugs are inactivated in dimethyl sulfoxide (DMSO). Thus drug discovery platforms that use it as a solvent are not able to study platinum drugs in combination with other drugs easily. Advances in Echo technology at the National Center for Advancing Translational Sciences (NCATS) enabled acoustic transfer of platinum drugs in aqueous solutions. This allows us to find novel small molecule/platinum drug combinations that can be tested in clinical trials.

**METHODS:**

We ran single-agent dose-response experiments to correctly estimate the dose-ranges needed for the matrix screens utilizing the OVCAR8 human carcinoma cell line. Then, we conducted our pilot matrix screen with compounds that are commonly combined with cisplatin for HGSOC treatment. Lastly, we spotted cisplatin against the Mechanism Interrogation PlatE drug library to identify novel combinations for clinical use against HGSOC. Compounds that show synergistic cell killing will give insight into novel drug combinations to treat HGSOC.

**RESULTS:**

Several synergistic and antagonistic effects were found. These results were refined with smaller-scale screening and tested in other HGSOC cell lines.

**CONCLUSION:**

The novel combinations discovered will allow chemotherapy patients to receive lower dosages of platinum drugs while maintaining or improving efficacy of cytotoxicity of cancer cells. This will help alleviate the side-effects of platinum drugs experienced by HGSOC patients.

